# Møller–Plesset Adiabatic Connection at Large Coupling Strengths for Open-Shell Systems

**DOI:** 10.1021/acs.jpca.4c00788

**Published:** 2024-05-08

**Authors:** Kimberly J. Daas, Eveline Klute, Michael Seidl, Paola Gori-Giorgi

**Affiliations:** † Department of Chemistry & Pharmaceutical Sciences and Amsterdam Institute of Molecular and Life Sciences (AIMMS), Faculty of Science, Vrije Universiteit, De Boelelaan 1083, Amsterdam 1081 HV, The Netherlands; ‡ Microsoft Research AI for Science, Evert van de Beekstraat 354, Schiphol 1118 CZ, The Netherlands

## Abstract

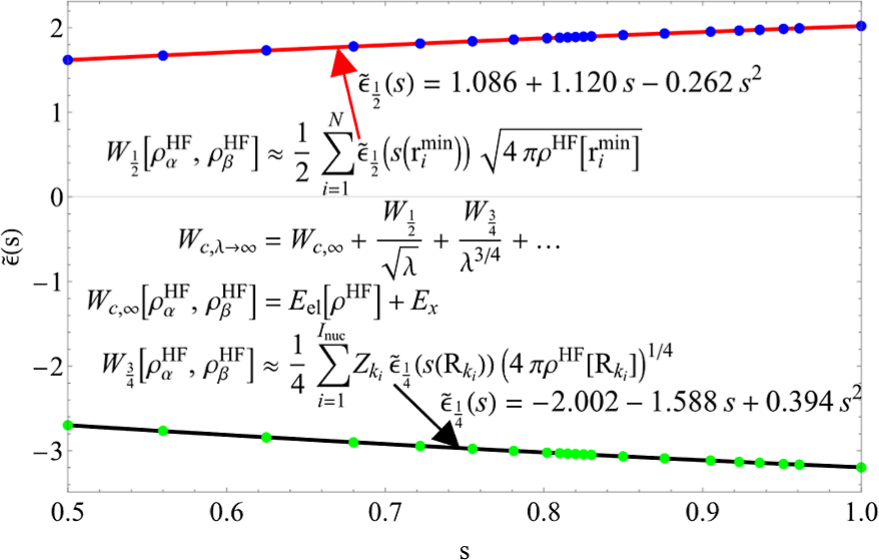

We study the adiabatic connection that has as weak-coupling expansion the Møller-Plesset perturbation series, generalizing to the open-shell case previous closed-shell results for the large-coupling limit. We first focus on the hydrogen atom with fractional spins, providing results along the adiabatic connection from small to large coupling strengths. We reveal an intriguing phase diagram and an equation for the large-coupling leading order that has closed-form solutions for specific choices of its relevant quantum numbers. We then show that the hydrogen atom results provide variational estimates for the large-coupling leading terms for the general many-electron open-shell case in terms of functionals of the Hartree–Fock α-spin and β-spin densities.

## 1 Introduction

Understanding and being able to compute the effects of spin within any approximate many-electron framework play a crucial role in the development of new quantum chemical methods.^1−13^ Already in the simple hydrogen molecule, spin plays an important role, ranging from restricted Hartree–Fock (RHF) having the wrong behavior for the total energy in the dissociation limit to unrestricted Hartree–Fock breaking the spin symmetry to fix it, and it also provides a paradigmatic case to understand static correlation errors of approximate density functional theory (DFT) functionals.^12−20^ The so-called flat plane conditions^12,13,15−18,20^ that can guide the construction of approximate functionals^21−23^ were derived by using the prototypical example of the spin dependence in the H atom.

Recently, the strong-coupling limit of the adiabatic connection (AC) that links the Hartree–Fock (HF) system to the physical one with the Møller–Plesset (MP) perturbation series as its weak coupling limit has been studied in detail, giving the exact result for the leading order term and variational estimates for the next two orders in the closed-shell case.^20,24−29^ All three of these terms are functionals of the HF density, ρ^HF^, only. Using these results, functionals that interpolate between the weak and strong coupling limits of the Møller–Plesset adiabatic connection (MPAC) have been introduced using the exact HF exchange and MP2 correlation energies combined with the strong-coupling limit.^30^ These interpolated functionals have been shown to massively improve MP2 interaction energies for a wide variety of noncovalent interactions, ranging from small charge-transfer complexes to larger π–π-bonded complexes.^30^ Generalizations to include other variants of MP2, such as opposite-spin only and regularization, have been found to be even more accurate at a lower computational cost.^31^

All these interpolation ideas originated from the DFT AC, where, instead, the Kohn–Sham system is connected to the physical system, and the strong coupling limit is given by the strictly correlated electron state.^29,32−45^ A fundamental difference between the two ACs is that in the DFT one, the density remains fixed as the coupling constant λ is turned on, whereas in the MPAC, the density can roam freely. In the DFT AC, the role of the spin state has been found to enter in the large coupling limit only at orders^36,41,46^, which means that it can be ignored in the two leading λ → ∞ terms used in the interpolating functionals.

However, in the MPAC case, the spin state already affects the second leading term at strong coupling due to the role of the exchange operator at this order and the lack of the density constraint.^20,26^ This spin-dependence becomes easy to study in the closed-shell case, where it has been shown^26^ that the result for the H atom with  spin-up and  spin-down electrons (denoted H) provides a variational estimate for the general many-electron case. However, this unnecessarily restricts the chemical space for which the new MPAC functionals can be used. Studying how the spin affects the MPAC strong coupling limit and also its role along the whole AC path beyond the closed-shell case is the gap that we fill in this work.

The paper starts with an introduction of the MPAC in Section 2, including a summary of previous results for its strong coupling limit. Since the closed-shell many-electron case was obtained by generalizing the result for H, we start by studying the H atom MPAC beyond the spin-unpolarized case in Section 3, where we derive and solve numerically the relevant equations, revealing an interesting phase diagram along the AC path. The spin-dependence of the strong-coupling limit coefficients is then extracted in Section 4. As we shall see, this limit defines an equation that has closed form solutions only for some special values of its parameters (orbital angular momentum and spin). We then show in Section 5 that the results for the H atom with fractional spins provide a variational estimate for the strong-coupling MPAC functionals for the general many-electron case in terms of the HF α-spin and β-spin densities. Conclusions and perspectives are discussed in Section 6.

## 2 Møller–Plesset AC

For a system with *N*_α_ spin-up and *N*_β_ spin-down electrons in a given external potential *v*_ext_(**r**), a HF calculation amounts to minimizing the expectation value of the physical Hamiltonian over single Slater determinants only, yielding *N* = *N*_α_ + *N*_β_ occupied HF spin orbitals ϕ_*i*_^HF^(**r**,σ) = ϕ_*i*_^HF^(**x**), with the HF electron density and the α-spin and β-spin densities
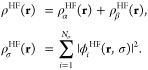
1

We keep the notation general, such that the following equations apply both to restricted (the spatial part of the α and β orbitals is forced to stay the same) and unrestricted open-shell HF.

Fixed in terms of these spin–orbitals (which are determined in the initial HF calculation), the standard Hartree and exchange operators  and  (that appear in the initial HF equations) do not change along the AC defined below. Subsequently, treating *Ĵ* and *K̂* as fixed (λ-independent) one-body operators, the MPAC for this N-electron system is represented by the generalized (λ-dependent) Hamiltonian
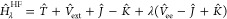
2where *T̂*, , and , respectively, are the kinetic energy, external potential *∑*_*i*_*v*_ext_(**r**_*i*_),^47^ and two-body electron–electron repulsion operators. Notice that  for λ ≠ 0 (and *N* ≥ 2) is no longer a true HF (one-body) Hamiltonian but includes a two-body interaction .

We denote the ground state of  by |Ψ_λ_⟩ and its corresponding eigenvalue by *E*_λ_^HF^
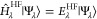
The Hellmann–Feynman theorem implies
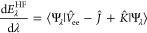
3

While  is the physical Hamiltonian (including the two-body interaction ), the one-body operator  is the original HF Hamiltonian whose ground state |Ψ_0_⟩ = |Ψ_λ=0_⟩ is the Slater determinant made of the occupied HF spin–orbitals {ϕ_*i*_^HF^}. The HF energy of the N-electron system is defined as 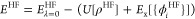
where the Hartree energy  is an explicit density functional
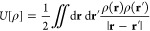
4while  is the usual HF exchange energy. The difference between the physical ground-state energy *E*_exact_ = *E*_λ=1_^HF^ and *E*^HF^ is the HF correlation energy
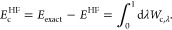
Here we have introduced the MPAC integrand
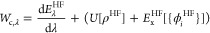
5where we have eq 3 for . The Taylor expansion of *W*_c,λ_ for λ → 0 is the MP perturbation series
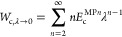
6

This expansion holds for closed systems. The first part of this work, however, addresses open fragments of larger systems (for example, an H atom within an infinitely stretched H_2_ molecule). In such cases, eq 6 applies to the whole system, while for the subsystem, we may find *W*_c,λ=0_ ≠ 0. An explicit example is reported in Figure 10 of ref (26), where it is shown that the MPAC result for the stretched H_2_ molecule tends to twice the result for the H atom with  spin-up and  spin-down as the distance *R* between the two H atoms increases, except at λ = 0, where the order of limits, *R* → ∞ and λ → 0, matters.

### 2.1 Previous Results on the λ → ∞ Expansion

The counterpart of eq 6 is the large coupling strength (λ → ∞) expansion^25,26^
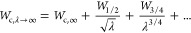
7For general N-electron systems (atoms or molecules with *M* fixed nuclear positions **R**_*k*_ with charge numbers *Z*_*k*_, where *k* = 1, ... , *M*), the leading term is^25^
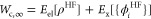
8Here, *E*_el_[ρ] is the classical electrostatic energy of *N* negative point charges (classical electrons) sitting at equilibrium positions in a rigid continuous positive background charge distribution with given density ρ(**r**)
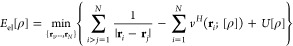
9with the electrostatic (Hartree) potential due to the charge distribution ρ(**r**)
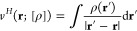


While the minimizing set {**r**_1_^min^,...,**r**_*N*_^min^} of equilibrium positions in eq 9 is typically not unique (depending on the symmetry group of the molecule), the set of *N* density values ρ^HF^(**r**_*i*_^min^), for *i* = 1, ..., *N*, should be unique. Independently, we expect a certain number *I*_nuc_ ≤ *N* of these positions **r**_*i*_^min^ to coincide with some of the fixed nuclear positions **R**_*k*_ (with *k* = 1, ..., *M*), implying that *I*_nuc_ ≤ *M*. Then, after relabeling the **r**_*i*_^min^ if necessary, we have
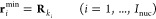
10In terms of these values ρ^HF^(**r**_*i*_^min^), the coefficients of the remaining terms in eq 7 have been shown^26^ to have the variational estimate for closed-shell N-electron systems
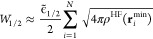
11
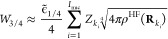
12

Here,  and  are the values labeled “” in Table 1.

**Table 1:** Value of  and  for the Hydrogen Atoms with Weight Factor *s* = 1 and  from ref (26)

	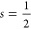	*s* = 1
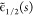	1.6185	2.0207
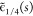	–2.70306	–3.2009

Eqs 11 and 12 for closed-shell systems were obtained in ref (26) by generalizing the exact coefficients for a closed-shell version  of the hydrogen atom
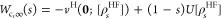
13
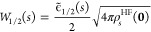
14
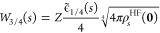
15

For the hydrogen atom (*N* = 1, , ), eq 9 has , and the only minimizing position **r**_1_^min^ = **0** coincides with the only nuclear position **R**_1_ = **0**, where minus the Hartree potential has its minimum. In ref (26), only the two cases *s* = 1 of a spin-polarized regular atom H[1,0] and  of a spin-unpolarized ensemble H were studied. The values of the three quantities ρ^HF^(**r**), , and  are *s* dependent, with the latter two for  reported again in Table 1 for completeness.

As a particular feature, the expansion (7) has a term *O*(λ^–3/4^). Such a term is absent in the corresponding λ → ∞ expansion for the density-fixed AC in DFT. According to eq 12, the term *O*(λ^–3/4^) in eq 7 occurs only in molecules with *I*_nuc_ > 0. For a short explanation,^26^ we note that the spatial probability distribution of the *N* electrons in the state |Ψ_λ_⟩ for λ → ∞ concentrates around the positions **r**_*i*_^min^. Consequently, each singularity  (with 1 ≤ *i* ≤ *I*_nuc_) of the external potential  in  of eq 2 contributes *O*(λ^1/4^) to *E*_λ_^HF^ as λ → ∞ and therefore *O*(λ^–3/4^) to *W*_c,λ_. Moreover, due to the Kato cusps of ρ^HF^(**r**) at nuclear positions, the term  in  for each *i* ≤ *I*_nuc_ produces two additional contributions *O*(λ^1/4^) to *E*_λ_^HF^. The result for the H case was proven to yield variational estimates for the many-electron closed-shell case.^26^

## 3 Møller–Plesset Adiabatic Connection for the H Atom with Fractional Spin

In this section, we generalize and compute the MPAC for the hydrogen atom at arbitrary values of  of the weight parameter *s*. This parameter *s*, defined in eq 17 below, must not be confused with the spin quantum number. In Section 5, we show that in the large-λ limit of the MPAC for a general open-shell system, the parameter *s* is linked to the local spin polarization, eq 54.

Here, we obtain results of the MPAC for λ ∈ [0, ∞) for the H atom with general *s*, which, as we shall see, reveal an interesting phase diagram.

### 3.1 HF Orbital ϕ_*s*_(**r**)

We consider a one-electron system  in a hydrogen-type external potential . Instead of being in a pure quantum state, however, this system is described by an ensemble of a spin-up state ϕ_α_(**r**)|α⟩ and a spin-down state ⟨ϕ(i)|α⟩ with weights of 1 – *w* and *w*, respectively. Statistically, our system has a spin
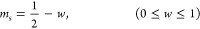
16

In this study, we stay in a restricted open-shell HF (ROHF) framework, forcing both spin states to have the same real spatial orbital ϕ(**r**) = ϕ_*s*_(**r**), which will depend on the weight *w* via^20^ the parameter *s*
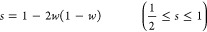
17and is fixed by minimizing the weight-dependent functional^20^
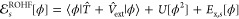


The influence of the restricted open-shell choice made here for the generalization to the many electron open shell case ofthe unrestricted case is discussed in Section 5.

Then, ϕ_*s*_(**r**)^2^ = ρ_*s*_^HF^(**r**) will be the HF density of eq 1, with the Hartree energy *U*[ρ_*s*_^HF^]. The exchange functional for this ensemble system is explicitly weight-dependent^20^
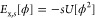


In the pure-state cases (*w* = 0 or *w* = 1), *E*_x,*s*_[ϕ] exactly compensates for the spurious Hartree interaction *U*[ϕ^2^]. In the ensemble case (0 < *w* < 1), this compensation is incomplete.^15,20^ This one-electron RHF functional of ϕ explicitly reads
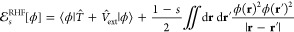
18In cases with *s* ≠ 1, the spherically symmetric (real-valued) minimizer
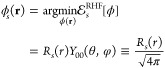
19will be different from the hydrogen ground state ψ_1*s*_(**r**). However, the radial wave function *R*_*s*_(*r*) is still finite at *r* = 0 and satisfies Kato’s cusp condition *R*_s_^′^(0) = −*ZR*_s_(0).^48^

The nonlinear self-consistent field Euler–Lagrange equation for ϕ_*s*_(**r**) corresponding to the minimization in eq 19
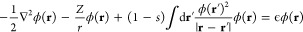
is solved using a basis set expansion in terms of Slater-type orbitals, as in ref (26)
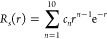
20

### 3.2 Møller–Plesset Adiabatic Connection: Equations and Numerical Solutions

Employing the HF orbital ϕ_*s*_(**r**), fixed by eq 19 for a given value of , we now consider the λ-dependent Hamiltonian of eq 2 for one-electron systems 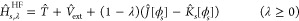
21

#### Operators *Ĵ* and 

3.2.1

For the present case of one-electron systems with fractional spin, the Hartree operator *Ĵ*[ϕ] and the (explicitly weight-dependent) exchange operator  are defined by their action
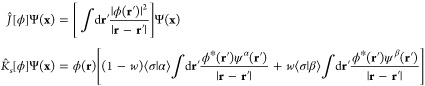
22on a general (single-particle) spin orbital
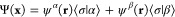


Notice that  is purely multiplicative, , with the weight-dependent Hartree potential *v*_*s*_^*H*^(**r**) = *v*^*H*^([ϕ_*s*_^2^];**r**)
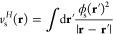
23while  is nonlocal and, in addition, acts on the spin variable σ in Ψ(**x**) = Ψ(**r**, σ). Equivalently, writing , the operator  can be defined via its kernel
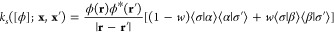
24

#### 3.2.2 Wave Function for Arbitrary λ > 0

In the case λ = 1,  is the physical Hamiltonian of the H atom, which has a spin-degenerate ground state. Any superposition of the kind
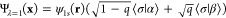
25where ψ_1*s*_(**r**) is the hydrogenic 1s orbital with *q* ∈ [0, 1] is a valid ground state. Alternatively, instead of the superposition, we can consider again a statistical ensemble.

Generalizing eq 25 to cases with arbitrary λ > 0, we write the ground state Ψ_*s*,λ_(**x**) of  as
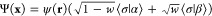
26where we have chosen *q* = *w*, forcing the spin expectation  in this pure state to equal the ensemble average . In other words, we suppress spin flip, and we stay on the restricted open-shell curve by enforcing the spatial orbital to be the same for both spins. We also notice that we could alternatively use an ensemble in eq 26 instead of a superposition. This does not change the result for the energy along the AC since the exchange kernel of eq 24, being diagonal in the spin part, yields the same expectation value for a superposition or an ensemble. All the equations reported below, in which the spin-dependence is explicitly transformed into a weight dependence in the MPAC Hamiltonian, are thus the same whether for the wave function at λ > 0, we use a superposition or an ensemble. The only constraint that matters is forbidding spin flips, which we enforce to keep the AC curve smooth. In fact, if we allow the spin to relax, the MPAC has a discontinuity^20,26^ as we cross λ = 1 (except in the case ). Since our aim is to build interpolations by using the information at large λ, we want to follow the AC that connects smoothly the λ ∈ [0, 1] region with the λ → ∞ limit.

With eq 26, the expectation of the Hamiltonian (21) can be written as
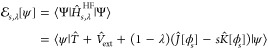
27

Here, we have performed the spin summation, turning  into the simpler operator *K̂*[ϕ], which no longer acts on spin
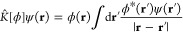
28but instead needs the weight parameter *s* from eq 17 as a prefactor in eq 27. As mentioned before, this holds regardless of the choice of using for Ψ(**x**) a superposition or an ensemble.

#### 3.2.3 Evaluation of the MPAC Integrand *W*_c,λ_

To evaluate the weight-dependent MPAC integrand of eq 5 for the present system
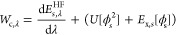
29we need the ground state energy *E*_*s*,λ_^HF^ of 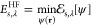
30

Since our (fixed) HF orbital  is spherically symmetric, the same is true for the Hartree potential in eq 23, *v*_s_^*H*^(**r**) = *v*_s_^*H*^(*r*), and the problem becomes block-diagonal in the orbital angular momentum . Therefore, the minimization (30) is performed separately for each , with the wave function , where (*r*) is the minimizer *u*(*r*), with  and *u*(0) = 0, in
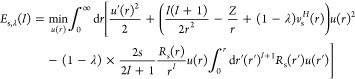
31

Then, the minimum (30) is obtained as
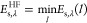
32

For any fixed value of *s*, the λ-dependent minimizer  = _s_(λ) can jump between different integers , as λ continuously grows from λ = 0 to λ = ∞. Plotted versus λ, *E*_s,λ_^HF^ will be continuous with possible kinks (and corresponding jumps in the derivative ).

The Euler–Lagrange equation for the minimization (31)
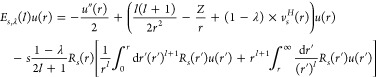
33is solved for a given {*s*, } pair with the spectral renormalization method,^49−52^ following the algorithm described in ref (26).

### 3.3 Results: Spin-Dependence along the MPAC

**Figure 1:**
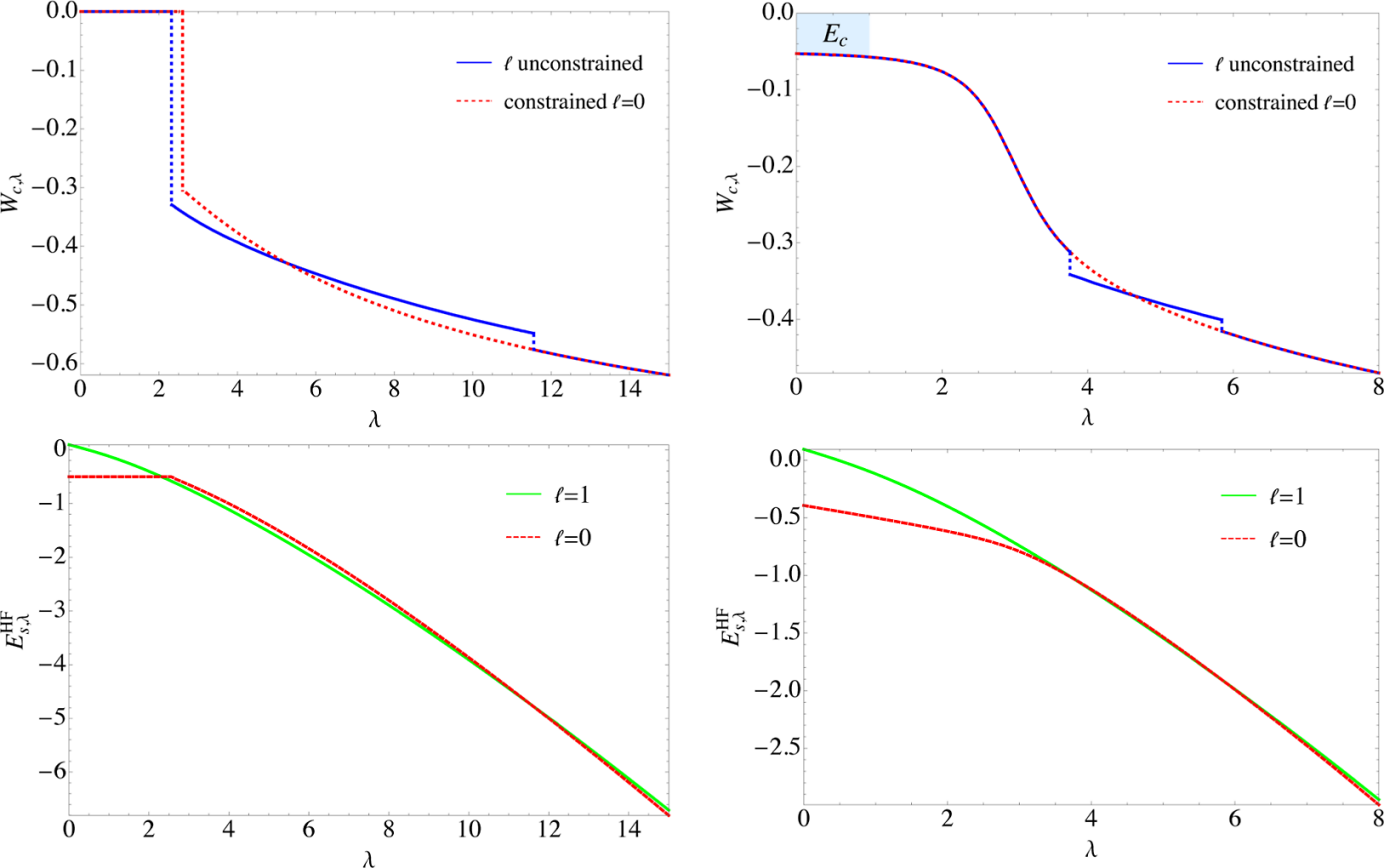
*W*_c,λ_ graph of *s* = 1 (*w* = {0, 1}) (top left) and *s* = 0.82 (*w* = {0.1, 0.9}) (top right) when  is unconstrained and when we constrain  = 0, with the blue shaded area being the correlation energy. The bottom two panels contain the corresponding  = 0 and  = 1 of the *E*_*s*λ_^HF^ curves, showing the crossings of states between the two channels.

In ref (26), it was found that for the spin-polarized (*s* = 1, or *w* = {0, 1}) H atom, the lowest energy *E*_*s*=1,λ_^HF^ starts at  = 0 for small λ, then a first crossing of states from  = 0 to  = 1 occurs at λ = 2.3, followed by a second crossing back to  = 0 around λ = 11.5, as shown here again on the bottom left panel of Figure 1. Instead, for the case , the  = 0 state was found to be the lowest for all λ ≥ 0. This means that the integrand *W*_c,λ_ has discontinuities in the case *s* = 1 (see the top left panel Figure 1), while it is continuous for .

The first question we address is thus whether the two crossings of states persist as we lower *s* from 1 to  and how they eventually disappear at . We find that, as we lower *s* starting from *s* = 1, the region of λ values for which  = 1 is the ground state shrinks, until it disappears entirely at *s* = 0.810.

**Figure 2:**
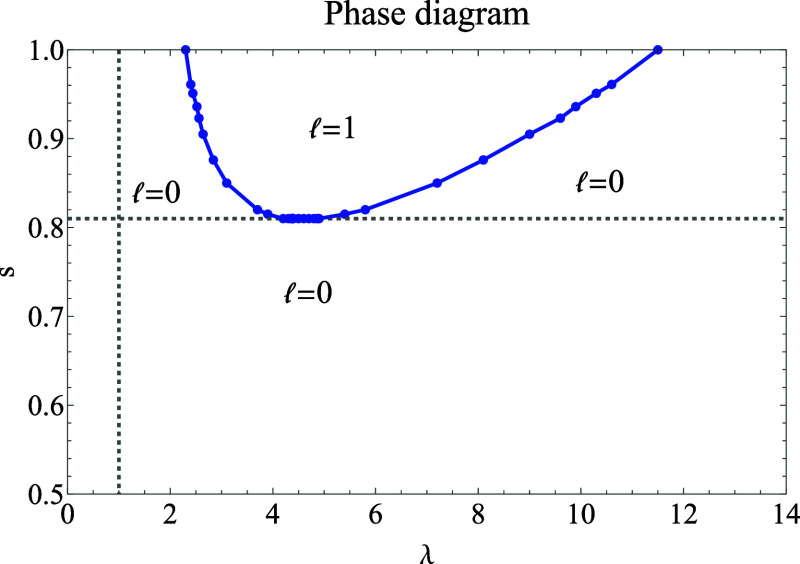
Phase diagram of *E*_*s*,λ_^HF^, showing in each region the angular momentum  corresponding to the lowest energy.

In Figure 2, we report the resulting phase diagram in the λ, *s* plane, showing the regions in which each  gives the lowest energy. We observe two distinct regions: the first, with 0.5 ≤ *s* ≤ 0.810 (0.1063 ≤ *w* ≤ 0.8937), has no crossing of states, with  = 0 being the ground state at all λ ≥ 0, which means that there are also no discontinuities in *W*_c,λ_. The second region, *s* > 0.810, has two crossings of states, with an intermediate λ range in which the  = 1 channel gives the lowest energy. The  > 1 channels have been found to always have higher energy in the physical range .

Similarly to constraining the spin, one can also follow the AC along the constrained  = 0 channel. This removes the crossings of states that introduce discontinuities in *W*_c,λ_; see, as an example, the right panels of Figure 1. Since the crossings always happen for λ > 1, interpolating between the large and small λ limits along the  = 0 curve allows us to make approximations of *W*_c,λ_ without affecting the resulting correlation energy. The only exception remains *s* = 1, which still has a discontinuity, this time at λ = 2.5 (see the left panels of Figure 1), due to a crossing between the flat 1s curve *E*_*s*=1,λ_^HF^ = −0.5 and the second  = 0 state, with a radial node. However, in all the other cases, the transitions are smooth, meaning that continuous curves for 0.5 ≤ *s* < 1 can be obtained when constraining  = 0.

## 4 λ → ∞ Coefficients for the H Atom

In this section, we compute the spin-dependence of the first three leading terms of the large-λ expansion of the H atom MPAC studied in the previous Section 3. We will then show in the next Section 5 that, similarly to the closed-shell case,^26^ these coefficients can be used in the general many-electron case.

We start from the Euler–Lagrange equation in eq 33. As λ → ∞, the effect of the term −λ*v*_*s*_^*H*^(*r*)*u*(*r*) becomes dominant:^26^ despite the presence of the quantum-mechanical kinetic-energy term and of the exchange operator, the solution *u*(*r*) will for λ → ∞ concentrate indefinitely at the minimum of −*v*_*s*_^*H*^(**r**) at **r** = **0**, implying
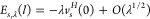
34To see this explicitly, eq 37 below, we expand^26^ in eq 33
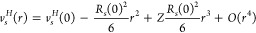
35
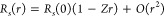
36where we have used the cusp condition *R*_*s*_^′^(0) = −*ZR*_*s*_(0) (see Section 3.1 above). We emphasize that *v*_*s*_^*H*^(*r*), in the hypothetic case *R*_*s*_^′^(0) = 0, would have no term *O*(*r*^3^). In terms of the scaled coordinate
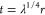
writing , with , eq 33 takes the form
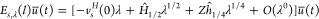
with λ-independent operators  and  given explicitly in ref (26). Here,  and  neither depend on *Z* nor on *v*_*s*_^*H*^(0) but still on *s*, , and (via the above expansions) also on *R*_*s*_(0). Writing
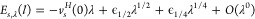
37we see that ϵ_1/2_ is the zero-order eigenvalue ϵ^(0)^ in the perturbation expansion for the operator 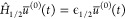
38while ϵ_1/4_ is the corresponding first-order correction ϵ^(1)^
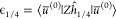
39

Via the operators  and , the values ϵ_1/2_ and ϵ_1/4_ depend on . This dependence is easily revealed
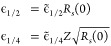
when we write , with  and . Then, eq 38 becomes a universal equation^26^ with two parameters,  and *s*
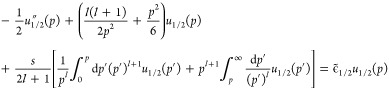
40

Numerically, the eigenvalue  is always lowest for  = 0, independently of *s*, confirming the results of the phase diagram of Figure 2. Therefore, for the determination of the large-λ coefficients, we can set  = 0 everywhere. Then eq 39 reads
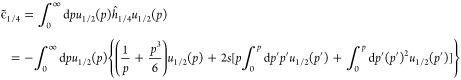
41

**Figure 3:**
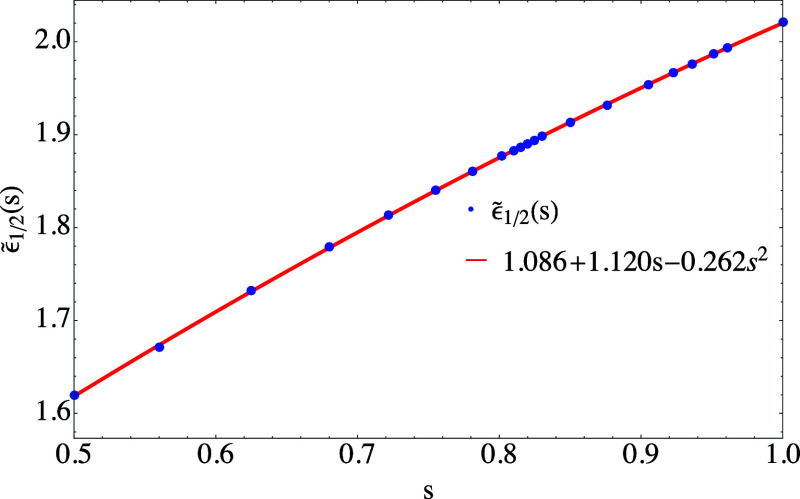
Dependence of  on *s* between 0.5 and 1 and a quadratic fit.

**Figure 4:**
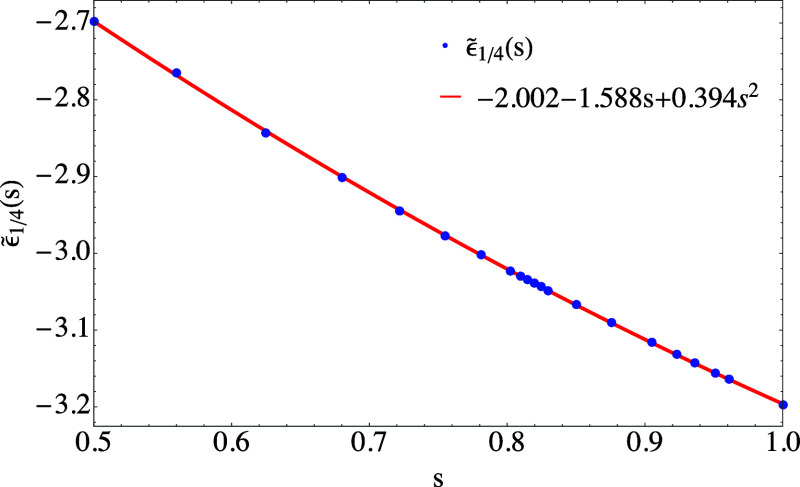
Dependence of  on *s* between 0.5 and 1 and a quadratic fit.

We then see that  and  for  = 0 are pure functions of *s*, see Figures 3 and 4.

In summary, eq 37 yields for  for large λ → ∞ (when the minimizer in eq 32 is always  = 0), the expansion of eq 7
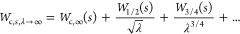
where the coefficients now depend on the weight parameter *s* = 1–2*w*(1 – *w*)
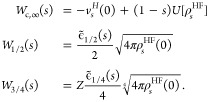


### Results: Functions  and 

4.1

To compute  and , we set  = 0 in eq 40 and expand *u*_1/2_(*p*) on the basis of the quantum isotropic harmonic oscillator (IHO) problem that arises if we set *s* = 0 in eq 40, which has frequency , and energies given by 
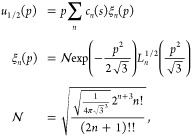
42with *L*_*n*_^1/2^ being generalized Laguerre polynomials.

Numerical solutions of eq 40 with  = 0 for different values of  have thus been obtained both by using the IHO basis set expansion of eq 42, which converges very fast, and by using the spectral renormalization method, finding perfect agreement for  and , which are shown, respectively, in Figures 3 and 4.

In both figures,  and  are accompanied by a quadratic fit, which proves to accurately interpolate the results and can be used for all practical purposes with maximum absolute deviations of 0.0026 and 0.0032, respectively.

In appendix A, we report an intriguing curiosity regarding eq 40: it has closed-form solutions for special pairs of *s* and  values. It was already noticed in ref (26) that the case *s* = 1,  = 0 has a simple closed-form solution, and in appendix A, we investigate the structure of eq 40 further, finding an infinite set of such solutions. Unfortunately, they all appear at *s* > 1 and thus have no physical meaning in the context we are analyzing.

## 5 General Many-Electron Case

We show in this section that the λ → ∞ results for the H atom at  provide a variational estimate for the strong-coupling leading terms of the general many-electron case in terms of functionals of the HF α-spin and β-spin densities.

The derivation is a generalization of the one for the spin-unpolarized (closed-shell) case:^26^ we start from a variational ansatz for the wave function Ψ_λ→∞_ that minimizes the Hamiltonian (2) when λ → ∞. We use a simple Hartree product of localized orbitals around each minimizing position **r**_*i*_^min^ of eq 9, with each spin in a superposition (again, for all the expectation values we need to consider in the derivation, it is the same if we use an ensemble for the spin part instead of a superposition). The antisymmetry of the wave function can be neglected as it contributes to orders  to the energy.^25,26,36^ The Hartree product then reads
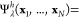
43
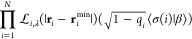
where
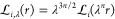
44 is a localized, normalized, 3D spherical function
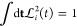
45which needs to be determined variationally, 0 ≤ *q*_*i*_ ≤ 1, and we will set later .^25,26^ In other words, we know that the wave function squared |Ψ_λ→∞_|^2^ tends to be a product of delta functions centered around the minimizing positions **r**_*i*_^min^, and with our ansatz, we seek the best variational spherical representation of the delta function that minimizes the next leading term. This approach does not take into account the coupling between the localized states and their anisotropy. As such, it can only provide a variational upper bound for the λ → ∞ functionals. The same kind of approximation was used by Wigner^53^ to compute the zero-point energy in the low-density electron gas, yielding an error of ∼12% with respect to the full coupled exact solution,^54^ which could provide an indication on the tightness of the upper bound we provide.

We thus evaluate the expectation of the Hamiltonian  of eq 2 on Ψ_λ_^*h*^, and we retain only the leading orders at λ → ∞. The kinetic energy and  are spin-independent: their expectation values at large λ are the same as for the closed-shell case considered in ref (26), which we report here again for completeness
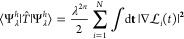
46and
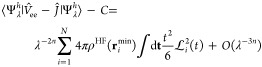
47where *C* = *E*_el_[ρ^HF^] – *U*[ρ^HF^].

The expectation of *K̂* is the only part that changes with respect to the closed-shell case. The kernel of *K̂* for a general open-shell system with *N*_α_ spin-up electrons and *N*_β_ = *N* – *N*_α_ spin-down electrons reads
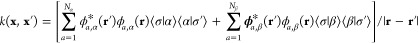
48and its expectation on Ψ_λ_^*h*^ at large λ is then
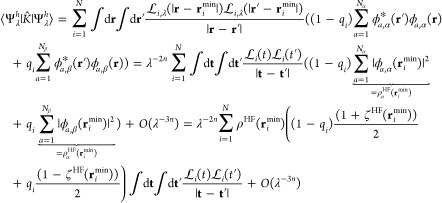
49where we have expanded the HF spin–orbitals ϕ_*a*,σ_ in scaled coordinates **t**_*i*_ = λ^*n*^(**r**_*i*_-**r**_*i*_^min^) at large λ
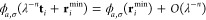
50and we have introduced the usual spin-polarization parameter for the HF α-spin and β-spin densities
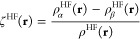
51

Now we see that the local spin polarization at the minimizing positions plays exactly the same role as *w* in our derivation for the H atom, with  and . If we want to use the λ → ∞ expansion to build interpolations, we need to forbid spin flip (to keep *W*_*c*,λ_ continuous) and thus set
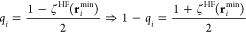
52With this choice, the leading (λ^–2*n*^) term of eq 49 becomes
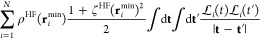
53We thus see that if we set
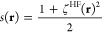
54(which varies between  and 1, exactly as in Section 3), we insert eqs 46, 47 and 53 in the expectation of  of eq 2 and set *n* = 1/4, we obtain, neglecting orders λ^1/4^ and lower,
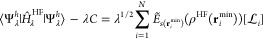
55where
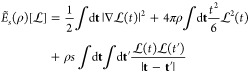
56

Exactly as in the closed-shell case,^26^ varying  with respect to  (keeping the normalization constraint), switching to the function , and introducing the scaled variable *p* = (4π ρ)^1/4^*t*, we obtain eq 40 with  = 0. This means that the best possible spherical variational ansatz for  is the same as the one we found for the *s*-dependent H atom in Section 3, around each equilibrium position **r**_*i*_^min^, yielding the variational estimate
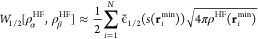
57where we can use for  the quadratic fit of Figure 3, and *s*(**r**) is a functional of the HF α-spin and β-spin densities via eqs 51 and 54.

The next leading order works exactly as in the closed-shell case.^26^ Its generalization to the open-shell case is then
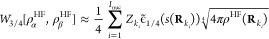
58where the sum runs only over the nuclear positions that coincide with minimizing electronic positions **r**_*i*_^min^, as in eq 10. For the function , we can use the quadratic fit of Figure 4.

We notice that these variational estimates are strictly valid for the restricted open-shell HF case only. For the unrestricted case, there would be an additional dependence on the α-spin and β-spin densities entering when we solve the *s*-dependent equation for the H atom, as the pair ϕ_*s*,α_(**0**) and ϕ_s,β_(**0**) appear in those equations, and the resulting functions  and  will be slightly different. However, we may expect these effects to be much smaller than the main *s*-dependence studied here (see, e.g., Figure 1 of ref (20)).

## 6 Conclusions and Perspectives

We have extended the results^26^ for the large-coupling limit of the AC that has as small-coupling expansion the MP series to the open-shell case. We first studied the paradigmatic case of the H atom, revealing an interesting phase diagram (Figure 2), and then showed that the results for the H atom at large coupling strength can be used for the general many-electron open shell case (Section 5), yielding functionals of the HF α-spin and β-spin densities.

Using these results, we plan to extend the construction of MPAC functionals^30,31^ to open shell systems, either by developing generalized gradient approximations for the leading term functionals, as done for the closed-shell case,^27^ or by using inequalities and relationships with the DFT AC case.^30,31^

## Appendix A

### Closed-Form Solutions for Special Values of *s* at = 0

We start by noticing that the energy, , for *s* = 1 and  = 0 is exactly equal to the energy of the first excited state of the isotropic harmonic oscillator, which arises by setting *s* = 0 in eq 40. Furthermore, the first excited state of *s* = 1 is again equal to the energy of the second excited state of *s* = 0 and has as a closed-form solution a linear combination of the ground state and the first two excited states. In summary, the energies of *s* = 1 and *s* = 0 are related, and their structure is shown in the first three columns of Table 2. Notice that in order to yield an energy of  for *s* = 1, one needs to mix in all IHO’s orbitals until *n*.

**Table 2:** Structure of the Closed-Form Solutions of eq 40, Where the *c*_*n*_’s of Every Combination of  and *s* are Different

	*s* = 0	*s* = 1	*s* = 10/3	*s* = 7	*s* = 12	*s* = 55/3
	*c*_0_ξ_0_					
	*c*_1_ξ_1_					
	*c*_2_ξ_2_					
	*c*_3_ξ_3_					
	*c*_4_ξ_4_					
	*c*_5_ξ_5_					

One can find other values of *s* that give closed-form solutions to eq 40 at  = 0. The next one, , is degenerate with the second excited state of the IHO (*s* = 0), , and is again a linear combination of the ground state and the first two excited states at *s* = 0. Closed-form ground- and excited-state energies for *s* = 7, *s* = 12, and  can also be obtained, see Table 2. One can find a formula for the values of these special *s*_*m*_ and the corresponding energies

A1

and

A2

with , and *n* increasing the excitation rank. The corresponding wave functions for these values of *s* are

A3

with

Another way to look at these results is to start from the full 3D version of eq 40 before the partial wave expansion, namely,

A4

We can then expand ψ_λ_(**p**) = *∑*_*n*_*c*_*n*_ϕ_*n*_(**p**), with ϕ_*n*_(**p**) the 3D IHO orbitals

A5

It can then be shown that the integral in the second term has the following expansion in terms of the *n* – 1 states

A6

Substituting equation into eqs A6 into A5 immediately results in the following condition to equate the terms with the error functions

A7

with *A*_*n*_ = *∫*ϕ_*n*_(**p**)d**p** and *b*_q_^(*n*)^ being a constant that depends on *n*. The remaining terms result in a second condition that can be written as

A8

The inner sum on the left-hand side only goes to *n* – 1, meaning that there are no terms of ϕ_*m*_ on the right-hand side. This adds a third condition on the energy, *E* = ϵ_*m*_, which proves eq A2.

Finally, a physical meaning of the closed-form solutions can be obtained by Fourier-transforming eq A4

A9

By using the scaled coordinate **y** = **k**/(4π)^1/4^ and multiplying both sides by 3, we have

A10

where . This equation describes the isotropic harmonic oscillator at , with the exchange integral transformed into a centrifugal potential that increases the angular momentum in Fourier space. The analytical solutions appear at the values of *s* for which the total angular momentum (kinetic plus exchange-induced) is an integer, yielding the back condition (A1) for  = 0. The fact that the λ → ∞ limit exchange contributes to the angular momentum in Fourier space also makes it clear why it enters at the same order (λ^–1/2^) as the kinetic energy.
